# PIKfyve Regulation of Endosome-Linked Pathways

**DOI:** 10.1111/j.1600-0854.2009.00915.x

**Published:** 2009-04-21

**Authors:** Jane de Lartigue, Hannah Polson, Morri Feldman, Kevan Shokat, Sharon A Tooze, Sylvie Urbé, Michael J Clague

**Affiliations:** 1 Secretory Pathways Laboratory, Cancer Research UK London Research Institute44 Lincoln's Inn Fields, London WC2A 3PX, UK; 2 University of California San Francisco, Cellular and Molecular Pharmacology600 16th Street, MC 2280, San Francisco, CA 94158-2280, USA; 3 Physiological Laboratory, School of Biomedical Sciences, University of LiverpoolCrown St., Liverpool, L69 3BX, UK

**Keywords:** autophagy, EGF receptor, endocytosis, phosphoinositide, PIKfyve

## Abstract

The phosphoinositide 5-kinase (PIKfyve) is a critical enzyme for the synthesis of PtdIns(3,5)*P*_2_, that has been implicated in various trafficking events associated with the endocytic pathway. We have now directly compared the effects of siRNA-mediated knockdown of PIKfyve in HeLa cells with a specific pharmacological inhibitor of enzyme activity. Both approaches induce changes in the distribution of CI-M6PR and trans-Golgi network (TGN)-46 proteins, which cycles between endosomes and TGN, leading to their accumulation in dispersed punctae, whilst the TGN marker golgin-245 retains a perinuclear disposition. Trafficking of CD8-CI-M6PR (retromer-dependent) and CD8-Furin (retromer-independent) chimeras from the cell surface to the TGN is delayed following drug administration, as is the transport of the Shiga toxin B-subunit. siRNA knockdown of PIKfyve produced no defect in epidermal growth factor receptor (EGFR) degradation, unless combined with knockdown of its activator molecule Vac14, suggesting that a low threshold of PtdIns(3,5)*P*_2_ is necessary and sufficient for this pathway. Accordingly pharmacological inhibition of PIKfyve results in a profound block to the lysosomal degradation of activated epidermal growth factor (EGF) and Met receptors. Immunofluorescence revealed EGF receptors to be trapped in the interior of a swollen endosomal compartment. In cells starved of amino acids, PIKfyve inhibition leads to the accumulation of the lipidated form of GFP-LC3, a marker of autophagosomal structures, which can be visualized as fluorescent punctae. We suggest that PIKfyve inhibition may render the late endosome/lysosome compartment refractory to fusion with both autophagosomes and with EGFR-containing multivesicular bodies.

3-phosphoinositides control the identity and dynamics of endocytic compartments, through the recruitment of multiple effector proteins containing specific recognition domains. PtdIns3*P* is generated by the class III PtdIns 3-kinase, hVps34, and is concentrated on early/sorting endosomes (1). It is required for receptor trafficking (2) and membrane fusion events (3), through its contribution to recruitment of several proteins containing FYVE or phox homology phox homology (PX) domains. The FYVE domain proteins early endosomal autoantigen-1 (EEA1) and hepatocyte growth factor regulated tyrosine kinase substrate (Hrs) are required for endosome fusion and receptor sorting respectively (4,5), whilst the PX domain containing Sorting Nexin 1 (SNX1) is a component of the retromer complex that mediates retrieval of the cation-independent mannose-6-phosphate receptor (CI-M6PR) from the endocytic pathway to the trans-Golgi network (TGN) (6).

PtdIns3*P* is also a precursor for the generation of PtdIns(3,5)*P*_2_ by the FYVE domain containing PtdIns 5-kinase, PIKfyve (7,8). The enzyme was first linked to the endosomal pathway by the observation that deletion of the yeast homologue results in markedly swollen vacuoles (9). Levels of PtdIns(3,5)*P*_2_ increase due to various stresses, osmotic shock in yeast (10) and ultraviolet (UV) radiation in mammalian cells (11). In yeast and mammalian cells PIKfyve interaction with Vac14 serves to stimulate enzymatic activity (12–14).

Identification of *bone fide* effectors of PtdIns(3,5)*P*_2_ has proven elusive (15). The best established is the yeast protein Svp1/Atg18, for which deletion results in a swollen vacuole phenotype (16). The mammalian homologues of Svp1 are the WD repeat domain containing, phosphoinositide-interacting 1 (WIPI-1) and WIPI-2 proteins. WIPI-1 (also known as WIPI-49) binds to PtdIns3*P* and PtdIns(3,5)*P*_2_, and altering its expression levels leads to changes in the distribution of CI-M6PR (17). Svp1/Atg18 was identified in a screen for yeast autophagy genes (18) and WIPI-1 has also been shown to associate with starvation-induced autophagic vacuoles (19).

Various studies have examined the effects of manipulating PIKfyve and Vac14 levels. A *Caenorhabditis elegans* (*C. elegans)* mutant hypomorphic for the PIKfyve homologue was suggested to possess a defect in retrieval of membrane from mature lysosomes (20). Deletion of *Drosophila* PIKfyve yields cells with enlarged endosomes and a defect in degradation of Wingless and Notch, without any apparent signalling defects (21). Vac14 −/− mice show a neurodegenerative defect, whilst at the cellular level, both large vacuoles and trapping of the CI-M6PR in endosomal compartments are evident (22). siRNA knockdown of PIKfyve is only partially effective, but also leads to defects in CI-M6PR trafficking whilst the degradation of epidermal growth factor receptor (EGFR) is unaffected (23).

Jefferies et al. have recently characterized a novel inhibitor of PIKfyve, YM201636, which provides the first opportunity for acute inhibition of the enzyme (24). This can allow discrimination of direct effects due to enzyme inhibition rather than longer-term adaptive responses of cells to knockout, or of protein functions unconnected to enzymatic activity. We now provide further characterization of the cellular effects of a PIKfyve inhibitor (MF4) pharmacologically similar to YM201636, which we have directly compared with knockdown of PIKfyve alone or in combination with Vac14. Our data reveal acute effects upon receptor tyrosine kinase (RTK) trafficking that reconcile with observations from model organisms, and provide new insight into PIKfyve involvement in cycling between TGN and endosomes as well as the autophagy pathway.

## Results

### PIKfyve inhibition creates swollen vacuoles inaccessible to fluid phase marker

**Figure 1:**
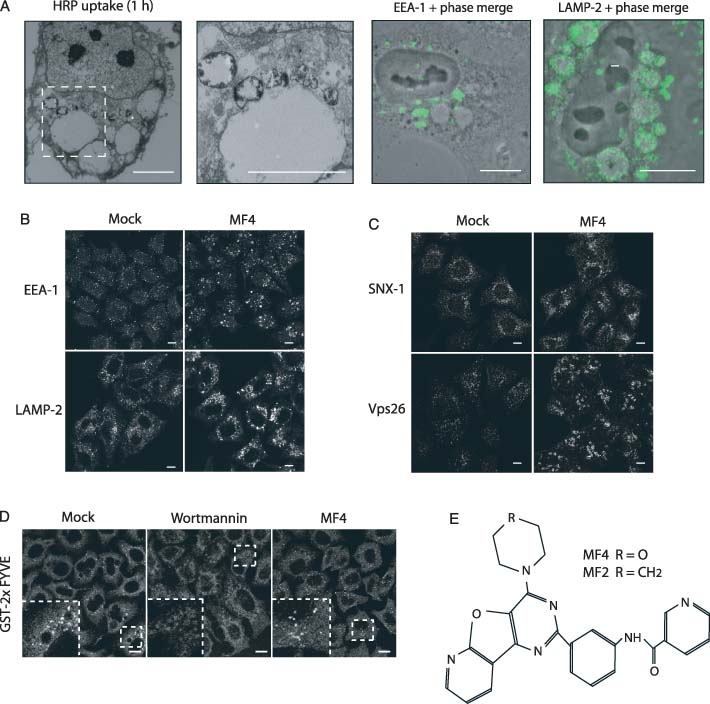
PIKfyve inhibition creates large swollen vacuoles inaccessible to fluid phase marker HeLa cells were treated with 800 nm PIKfyve inhibitor (MF4) for 4 h prior to either fixation and staining with a variety of markers by immunofluorescence, or uptake of HRP for 1 h, fixation and processing for EM (see *Materials and Methods* for details). Wortmannin was added to cells at 100 nm for 30 min. A) MF4-mediated inhibition of PIKfyve causes the formation of swollen vacuoles that are inaccessible to internalized HRP. These vacuoles are surrounded by a second class of smaller HRP-containing vacuoles. B) Both early and late endosomal markers are associated with distinct swollen endosomes that surround these vacuoles. C) Retromer components Vps26 and SNX-1 also associate with swollen endosomal compartments. Scale bars = 10 μm. D) The PtdIns 3-kinase inhibitor Wortmannin ablates endosomal PtdIns(3)*P* staining, whilst MF4 treatment has no significant effect. Scale bars = 20 μm. E) Chemical structure of MF4 (PIKfyve inhibitor) and MF2 (inactive analogue).

Knockdown of PIKfyve in HeLa cells creates swollen vacuoles visible by phase contrast light microscopy in ∼30% of cells as previously reported (23). We could obtain highly efficient knockdown of the PIKfyve activator protein Vac14 but this only produced the vacuole phenotype at very low penetrance (∼3%) and did not augment the effect of PIKfyve knockdown on vacuole formation (not shown). MF4 is chemically similar to the recently described specific PIKfyve inhibitor YM201636 by Jefferies et al., with the only difference being that MF4 lacks an amino group on the pyridine ring (24), (Figure 1E). MF4 inhibited PIKfyve with an IC_50_ of 23 nm, whereas an inactive analogue MF2 showed no activity even at 5 μm. Corresponding MF4 values for class I PtdIns 3-kinases which we determined are 0.25 μm (p110α), 1 μm (p110β), 0.9 μm (p110γ) and 0.8 μM (p110δ). Application of MF4 gives a vacuolar phenotype in all cells within 4 h. Electron microscopic analysis indicates that the large phase lucent vacuoles are inaccessible to internalized horseradish peroxidase (HRP), but they do become surrounded by a second class of smaller (but still swollen) HRP-containing vacuoles, positive for early (EEA1) or late endosomal markers [lysosome-associated membrane protein (LAMP-2)] (Figure 1A and B), which we will henceforth refer to as 'swollen endosomes’. The retromer components Vps26 and SNX1 also associate with swollen endosomal structures (Figure 1C). MF4 does not appreciably reduce cellular PtdIns3*P* levels or its cellular distribution, as assessed by immunofluorescence labelling of cells for this lipid with a GST-2xFYVE probe (Figure 1D).

### Disruption of endosome to TGN trafficking

**Figure 2:**
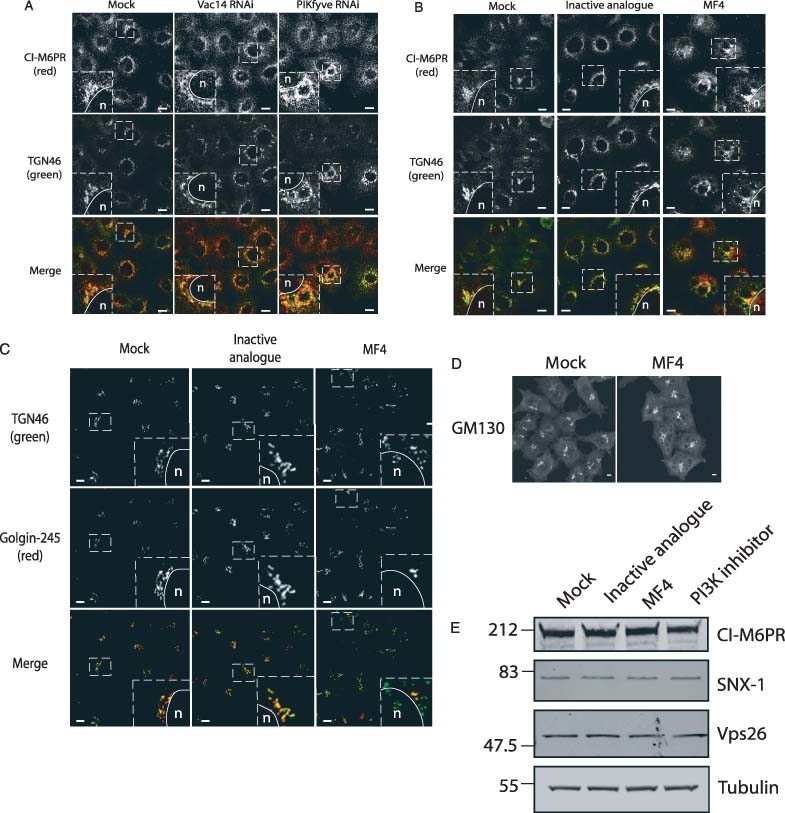
Inhibition of PIKfyve causes a disruption in steady-state distribution of ciM6PR and TGN46 HeLa cells were treated with 40 nM siRNA oligos for 72 h or 800 nm MF4 for 4 h. Both knockdown and inhibition of PIKfyve alter the steady-state localization of ciM6PR, whereas Vac14 knockdown and MF2 (inactive analogue of the PIKfyve inhibitor MF4) have no effect (A and B). The markers fragment into distinct vesicular compartments, which show little co-localization. The distribution of a second TGN resident protein golgin-245 is unaffected and retains its characteristic perinuclear localization (D). The *cis*-golgi marker GM130 also retains it's ribbon-like disposition (E). There is no change in the levels of ciM6PR or retromer components upon treatment with PIKfyve inhibitor (F). Scale bars = 20 μm.

PIKfyve has been implicated in the control of the retromer-mediated pathway between endosomes and the TGN through siRNA studies of CI-M6PR distribution with respect to the marker protein TGN-46 (23). Certain aspects of these studies may be complicated by the fact that TGN-46 itself undergoes a cycling itinerary, which partially overlaps with ciM6PR (25). We have now analyzed the steady-state distribution of TGN-46, CI-M6PR and golgin-245 following individual knockdown of PIKfyve, hVac14, a combination of the two and the PIKfyve inhibitor. Knockdown of PIKfyve or its inhibition leads to a more dispersed distribution of CI-M6PR but also of TGN-46, whilst knockdown of Vac14 has little effect (Figure 2A and B). In cells where the TGN46 has been highly dispersed, there is little co-localization with CI-M6PR or with an alternative TGN marker golgin-245 (26), which retains its characteristic perinuclear localization (Figure 2C). Under conditions where TGN-46 is highly dispersed, the *cis*-Golgi marker GM130 retains its ribbon-like disposition (Figure 2D), characteristic of intact Golgi structure. No changes in levels of CI-M6PR or of retromer components are seen as a consequence of PIKfyve inhibition (Figure 2E).

**Figure 3:**
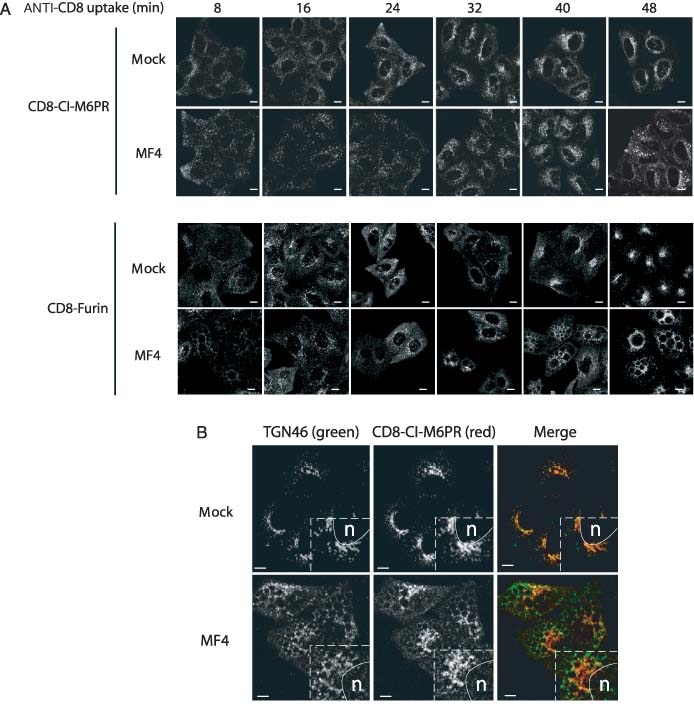
PIKfyve inhibition causes a delay in CD8-ciM6PR and CD8-Furin retrieval Uptake of CD8 antibody in HeLa M cell lines stably expressing CD8-ciM6PR and CD8-Furin was examined following treatment of cells with 800 nm MF4 PIKfyve inhibitor. Both Furin and ciM6PR retrieval to a trans-Golgi localization is delayed upon inhibition of PIKfyve (A). At the terminal uptake time-point, in cells where TGN46 is highly fragmented, there is negligible co-localization with CD8-ciM6PR (B). Scale bars = 20 μm.

**Figure 4:**
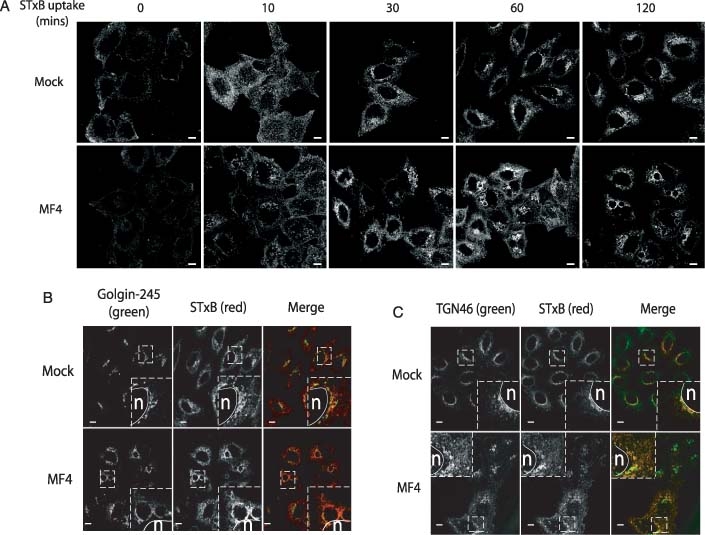
PIKfyve inhibition causes a perturbation in the uptake of the Shiga toxin B-subunit Uptake of the Shiga toxin B-subunit was examined in HeLa cells using a Cy3-labelled StxB construct, following treatment with 800 nm MF4 PIKfyve inhibitor. Cy3-STxB retrieval to a perinuclear localization is also delayed in cells treated with PIKfyve inhibitor (A), but ultimately reaches a TGN localization, as judged by co-localization with golgin-245 (B). In cells where TGN46 is highly dispersed, co-localization with Cy3-STxB can be observed, indicative of the fact that these two proteins may traffic on a similar retrieval pathway (C). Scale bars = 20 μm.

Cell surface exposed CI-M6PR can be retrieved to the TGN following endocytosis, and Shiga toxin follows an overlapping retromer-dependent route (27). We have followed trafficking of a chimeric CD8-CI-M6PR along this route following application of an anti-CD8 antibody and of fluorescently labelled Shiga toxin B-subunit. Furthermore, we have followed the trafficking of a CD8-Furin chimeric protein, which also traffics from the cell surface to the TGN, but in a retromer-independent fashion (28). Following PIKfyve inhibition, internalized CD8-CI-M6PR and CD8-Furin both can reach a TGN compartment, but show slower rates of accumulation at this perinuclear destination (Figure 3A). In cells which show highly dispersed TGN-46 punctae, these do not co-localize with CD8-CI-M6PR at the final time-point (Figure 3B). Shiga toxin can reach the perinuclear TGN as judged by co-localization with golgin-245, (Figure 4A and B) following PIKfyve inhibition, but its accumulation there, is impeded and co-localization with dispersed TGN-46 positive punctae can also be observed (Figure 4C).

### Downregulation of RTKs

**Figure 5:**
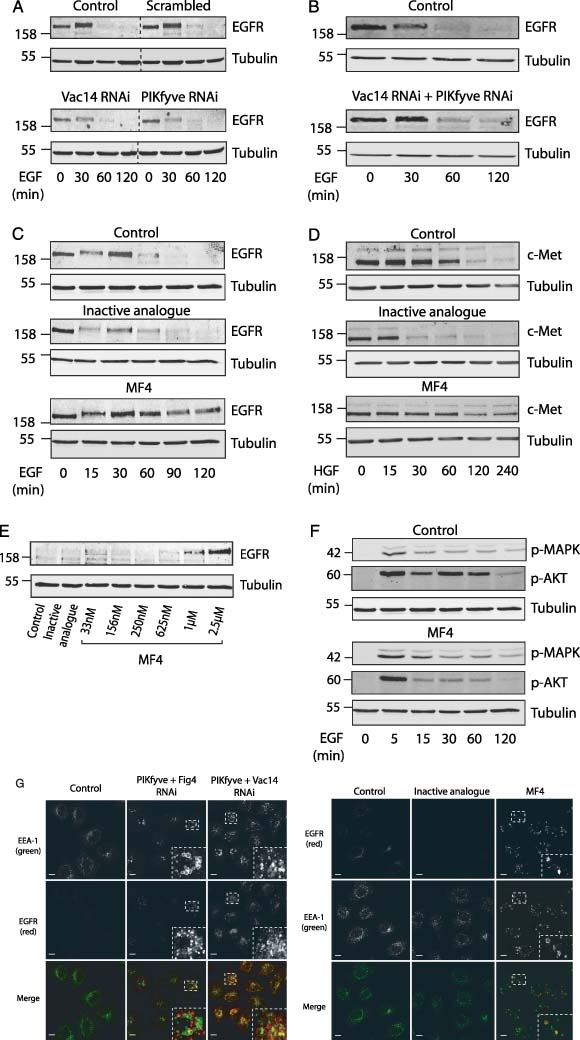
Acute inhibition of PIKfyve affects downregulation of tyrosine kinase receptors HeLa cells were treated with siRNA oligos against the indicated proteins (A and B), or with 3 μm PIKfyve inhibitor (C and D) or the indicated doses of inhibitor (E). Serum-starved cells were stimulated with 100 ng/mL EGF or HGF for the indicated times. A) Individual knockdown of PIKfyve or Vac14 did not affect the degradation of either EGFR or c-Met. B) A combined knockdown of PIKfyve with Vac14 caused a modest delay in receptor downregulation. C) Treatment with PIKfyve inhibitor caused a severe delay in EGFR. D) c-Met downregulation. E) Concentration dependence of MF4 on EGFR downregulation after 120 min EGF stimulation. F) pMAPK and pAKT signalling is not prolonged following treatment with PIKfyve inhibitor, pAKT signalling is dampened at later time-points. α-tubulin is used as a protein loading control. G) Immunofluorescence images demonstrating the accumulation of EGFR in PIKfyve inhibitor and combined knockdown treated HeLa cells. HeLa cells were transfected with 40 nM of the indicated oligos over 72 h, then serum-starved overnight, and following treatment with 800 nm PIKfyve inhibitor (in place of siRNA treatment; where indicated), were stimulated with 100 ng/mL EGF for 4 h. Following fixation with 3% PFA in PBS cells were labelled with anti-EGFR and anti-EEA-1. A proportion of the EGFR can be seen in the interior of swollen early endosomal compartments (G). Scale bars = 20 μm.

PIKfyve siRNA studies of EGF-induced EGFR downregulation have not revealed any defect in EGFR downregulation (23). Our data concur with these previous studies, but we noticed that when combined with Vac14 knockdown a modest defect in EGFR downregulation is evident (Figure 5A and B). A far more effective block to EGFR and also c-Met downregulation was seen following application of PIKfyve inhibitor (Figure 5C, D and E). Fluorescence examination of EGFR distribution shows that residual receptor following PIKfyve inhibition is concentrated in punctate structures which frequently co-localize with the early endosomal marker EEA1 (29). In many cases EGFR can be visualized in the interior of endosomes decorated with EEA1 on their limiting membrane (Figure 5G). Thus acute pharmacological inhibition has revealed an important function of PIKfyve in mammalian cells that provides some reconciliation with data obtained with *Drosophilia* knock-outs (21), which indicated defects in receptor downregulation. If activated receptor was retained at the limiting membrane of endosomes, one may expect more prolonged signalling following acute activation (30). We do not see this when we examine activation of mitogen-activated protein kinase (MAPK) or AKT/protein kinase B (PKB). Rather we see a dampening of AKT signalling at later time-points, e.g. 30 min (Figure 5F). Although IC50 values of MF4 (23 nm) and YM210636 (33 nm) are similar towards PIKfyve, we obtain less selectivity over PtdIns 3-kinase p110*α* than that reported for YM210636 (24). To be certain that the observed effects on EGFR degradation were not as a result of p110α inhibition, specific inhibitors for both p110*α* and *β* were used. Both inhibitors caused a significant reduction in phosphorylation of AKT/PKB but had no effect on EGFR degradation (data not shown). These results in combination with the observation that MF4 does not cause a reduction in initial EGF-dependent phosphorylation of AKT/PKB, suggest that the effects on EGFR degradation are not due to a non-specific inhibition of class 1 PtdIns 3-kinases.

### Role of PIKfyve in autophagy

**Figure 6:**
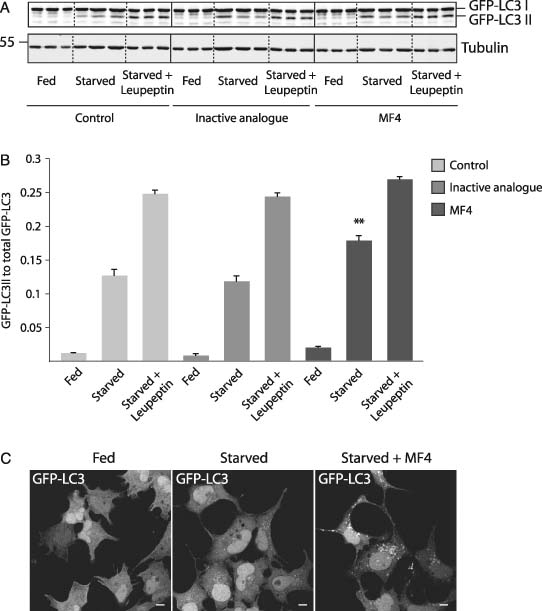
Inhibition of PIKfyve leads to an increase in starvation-induced GFP-LC3 lipidation HEK293A cells stably transfected with GFP-LC3 were treated with MF4 PIKfyve inhibitor or an inactive analogue (MF2) for 4 h prior to replacement of fresh growth medium (fed) or Earles’ balanced salt solution (EBSS) for 2 h (starved). Cells were subsequently lysed or processed for immunofluorescence. A) Western blot on triplicate samples indicating the levels of GFP-LC3 I and II in fed and starved cells upon treatment with MF4 and with or without the protease inhibitor Leupeptin. B) Quantitative analysis of the ratio of GFP-LC3 II to total GFP-LC3 from A. PIKfyve inhibition causes a statistically significant (*n* = 3, *p <* 0.01, T-test) accumulation of lipidated GFP-LC3 upon starvation-induced autophagy. C) Immunofluorescence analysis of GFP-LC3 II punctae formation following starvation-induced autophagy. The accumulation of GFP-LC3 II positive punctae can be observed following treatment with PIKfyve inhibitor. Scale bars = 20 μm.

PtdIns3*P* generated by the PtdIns 3-kinase Vps34 is required for initial steps of autophagosome formation, but the role of PtdIns(3,5)*P*_2_ is less well established. The Atg18 gene in yeast binds both 3-phosphoinositides with a preference for PtdIns(3,5)*P*_2_(16). One signature for the accumulation of autophagosomes is the enrichment of a faster migrating form (LC3 II) of the autophagy protein LC3, following its conjugation to phosphatidyl-ethanolamine (31). Whilst, neither Vac14 nor PIKfyve knockdown had any significant influence on starvation-induced accumulation of GFP-LC3-II (data not shown), inhibitor treatment led to a significant increase, as judged biochemically (Figure 6A and B) and by the visualization of GFP-labelled punctae (Figure 6C). Addition of the PIKfyve inhibitor, MF4 did not significantly increase the accumulation of GFP-LC3 in the presence of leupeptin, a condition in which the degradation of GFP in the late endosome/autolysosome should be reduced. This indicates that MF4 treatment does not increase the rate of formation of autophagosomes but instead interferes with their consumption.

## Discussion

Previous studies in mammalian cells have assessed phenotypes arising from siRNA knockdown. These have the following drawbacks; (i) PIKfyve has proven hard to completely deplete, so that residual enzymatic activity could provide enough PtdIns(3,5)*P*_2_ particularly if the enzyme is controlled by a positive feedback loop, (ii) it is not clear if phenotypes observed are because of loss of enzyme activity or of some other function of the protein and (iii) depletion is very gradual, so that any phenotype may constitute a very indirect consequence of protein loss over a prolonged time period. Nevertheless, these studies and our current study, have shown that the distribution of proteins which traffic between the sorting endosome and the TGN is perturbed (23). This result is recapitulated by acute pharmacological inhibition. One of these proteins is TGN-46, which, because it is also cycling along this pathway cannot be reliably used as a TGN marker under these conditions. Golgin-245, another TGN marker, shows significantly less perturbation following PIKfyve inhibition. Dispersed TGN-46 punctae produced by PIKfyve inhibition do not co-localize with golgin-245, suggesting that TGN-46 may accumulate in another compartment that forms part of its itinerary. Whilst Jeffries et al.suggest that the steady-state distribution of CI-M6PR is not drastically altered following treatment with YM201636, their experiments were performed in a different cell line (NIH3T3) and other assays for retrieval on this pathway were not examined (24). In accordance with our data, a recent study of Vac14 −/− mice showed a similar defect in CI-M6PR distribution (22).

As well as the steady-state distribution, the kinetics of transport from the cell surface via the endosome to the TGN is delayed for several proteins (CD8-CI-M6PR, CD8-Furin, Shiga Toxin), which may utilize retromer-dependent (CD8-CI-M6PR, Shiga toxin) and retromer-independent (CD8-Furin) sorting mechanisms from the endosome. Interestingly, retrieval to the TGN is delayed and not completely blocked following loss of PIKfyve activity, suggesting that either cargo may traffic on alternate, less efficient pathways or that enzymatic activity is rate-limiting for this process but not essential.

Knockdown of PIKfyve (23) or of Vac14 (this study) does not significantly affect EGFR degradation rates, consistent with previous studies. This observation has been difficult to reconcile with knockout phenotypes in model organisms which have suggested a critical role for PIKfyve in receptor degradation and formation of terminal lysosomes (20,21). We reasoned that in the circumstance of incomplete depletion, combining Vac14 and PIKfyve knockdown should suppress PtdIns(3,5)*P*_2_ to a greater extent than PIKfyve alone. This condition revealed a modest inhibition of EGFR degradation (Figure 4B), suggesting a role for PtdIns(3,5)*P*_2_ synthesis, but with a relatively low threshold value. Consistent with this observation, pharmacological inhibition of PIKfyve, at concentrations which do not produce any major perturbation in the levels of other 3-phosphoinositides, revealed a strong block to both EGFR and c-Met downregulation, as judged by western blotting and by immunofluorescence. The appearance of EGFR in the interior of swollen endosomes positive for EEA1 suggests that the major impediment is not a defect in sorting to luminal vesicles, but may reflect a failure of multivesicular bodies to fuse with the degradative late endosome/lysosomal compartment.

A role for PtdIns3*P* in the formation of autophagosomes is well established (32), but the part played by PtdIns(3,5)*P*_2_ is less clear. We observe that PIKfyve inhibition leads to an accumulation of lipidated GFP-LC3 on punctate structures, characteristic of autophagosomes (33). This could reflect an increased rate of production of autophagosomal structures or a decreased rate of their consumption by fusion with lysosomes. Taking this data together with the observation of Jefferies et al., that autophagic breakdown of proteins is reduced by PIKfyve inhibition (24), we favour the second interpretation. Thus, both effects on the autophagy pathway and of EGFR downregulation could be explained by a common mechanism, through which PIKfyve inhibition either renders an acceptor late endosome/lysosome compartment refractory to fusion or leads to a defect in the acidification necessary for lysosomal degradation. Although not quantitative, Acridine Orange accumulation in endocytic punctae, indicative of acidification, is similar in both MF4-treated and control cells, although it does not accumulate in the highly swollen vacuoles which appear disconnected from the endocytic pathway (data not shown). Two general models can explain how PtdIns(3,5)*P*_2_ effects are mediated; (i) by recruitment of effector molecules with specific lipid binding domains and (ii) allosteric regulation of resident endocytic proteins, e.g. enzyme or channel activity. Although Svp1 in yeast is an authentic PtdIns(3,5)*P*_2_ sensor it is less clear for its mammalian orthologues WIPI-1 and WIPI-2. WIPI-1, like Svp1 can bind both PtdIns3*P* and PtdIns(3,5)*P*_2_*in vitro*(17) whilst the lipid binding-properties of WIPI-2 are unknown. Other candidate effector proteins remain to be identified.

In summary, juxtaposition of siRNA knockdown of PIKfyve with pharmacological inhibition links the observed siRNA phenotype of disrupted endosome to TGN trafficking, with enzymatic activity. However, pharmacological inhibition reveals further defects on the receptor degradation and autophagy pathway, suggesting that relatively low levels of PtdIns(3,5)*P*_2_ are permissive for these pathways.

## Materials and Methods

### Antibodies and other reagents

Rabbit polyclonal human Vac14 antibodies were raised by immunizing rabbits with the N-terminal amino acids of Vac14 (HLEVRHQRSGRGDHLDRR), conjugated via a cysteine to *Limulus polyphemus* haemocyanin (Covance) and affinity purified on peptide columns. Sheep anti-TGN46, sheep anti-GM130 antibodies and sheep anti-GFP were generous gifts of Vas Ponnambalam (University of Leeds, UK) Francis Barr and Ian Prior (both University of Liverpool, UK) respectively. Rabbit polyclonal anti-PIKfyve and anti-CI-M6PR antibodies were generous gifts from Lois Weisman (University of Michigan, USA) and Paul Luzio (Cambridge Institute of Medical Research, UK). Mouse monoclonal anti-Met (25H2) antibody, rabbit monoclonal phosphorylated AKT (pAkt) (Ser 473) and mouse monoclonal phosphorylated MAPK (pMAPK) (Thr 202, Tyr 204) antibodies were from Cell Signalling. Monoclonal anti-EGFR R1 was from Cancer Research UK Laboratories (CRUK) and goat polyclonal anti-EGFR 1005 were from Santa Cruz. Mouse monoclonal anti-tubulin was from Sigma. Mouse monoclonal anti-golgin 245 (p230) antibody was from BD Biosciences. Rabbit polyclonal anti-EEA-1 was previously described (4). Secondary antibodies were from Molecular Probes and Licor Biosciences. HeLa M cell lines stably expressing CD8-CI-M6PR and CD8-Furin along with mouse monoclonal CD8 antibody were generous gifts from Matthew Seaman (Cambridge Institute of Medical Research, UK). Purified mouse EGF was obtained from J. Smith, Liverpool, UK. 2GL9 cells, HEK293A cells stably transfected with GFP-LC3 have been previously described (34). GST-2xFYVE (Hrs) vector was a gift from Harald Stenmark (35) Following production in bacteria it was isolated with Glutathione Sepharose and then biotinylated with Sulfo-NHS-LC-Biotin (Pierce, UK). Purified PIKfyve protein was a kind gift from Frank Cooke (UCL, UK).

### Synthesis and characterization of MF4 and MF2

Synthesis of the PIKfyve inhibitor (hereafter designated MF4) and its inactive analogue, MF2 is described below. PI-103 (36) (200 mg, 0.57 mmol) and 2,6-di-tert-butyl-4-methylpyridine (136 mg, 0.66 mmol) were dissolved in CH_2_Cl_2_ (20 mL) and cooled on ice. Triflic anhydride (0.3 mL, 0.63 mmol) was added drop-wise and the reaction was warmed to room temperature. Further CH_2_Cl_2_ was added (10 mL) and the reaction was allowed to proceed overnight. The solvent was removed *in vacuo* and the product was purified by silica gel flash chromatography with 25% EtOAc:Hexanes to yield 67 mg (24% yield). Liquid chromatography–electrospray ionisation–mass spectrometry (LC-ESI-MS) [MH]^+^ m/z calculated for C_20_H_15_F_3_N_4_O_5_S 481.1, found 480.9. This product (67 mg, 0.14 mmol), cesium carbonate (64 mg, 0.20 mmol) and nicotinamide (21 mg, 0.17 mmol) were added to dioxane (4 mL). Xantphos (10 mg, 0.016 mmol) and Pd_2_(dba)_3_ (5 mg, 0.006 mmol) were added and the reaction was heated to 100°C for 24 h (37). The reaction was diluted with CH_2_Cl_2_, filtered and the solvent was removed *in vacuo.* The product was purified by silica gel chromatography using a gradient of 2–3% MeOH:CH_2_Cl_2_ and by reverse phase high performance liquid chromotography (HPLC) using a MeCN/H_2_O/0.1% trifluoroacetate (TFA) solvent system to give a white solid. LC-ESI-MS [MH]^+^ m/z calculated for C_25_H_20_N_6_O_3_453.2, found 453.0. MF2 was prepared similarly using PIK-112 (36) in place of PI-103. LC-ESI-MS [MH]^+^ m/z calculated for C_26_ H_22_N_6_O_2_451.2, found 451.4.

### In vitro kinase assay

Purified GST-PIKfyve bound to 5 μL of glutatione beads was incubated with inhibitors at fourfold dilutions over a concentration range of 5 μm to 0.001 μm in a total reaction volume of 25 μL. The assay buffer contained 10 μm ATP, 2 μCi of (γ-32P) ATP, 100 μm PI-3P, 400 μm phosphatidyl-ethanolamine, 25 mm HEPES (pH 7.4), 120 mm NaCl, 1.5 mm MgCl_2_, 1 mm DTT, 0.5 mg/mL BSA and 2% dimethyl sulphoxide (DMSO). Reactions were terminated by spotting onto nitrocellulose membranes, which were then washed five or six times to remove unbound radioactivity and dried. Transferred radioactivity was quantitated using phosphorimaging, and IC50 values were calculated by fitting the data to a sigmoidal dose-response using Prism (GraphPad).

### Cell culture, PIKfyve inhibitor treatment and siRNA transfection

HeLa and HEK293A cells were cultured in 5% CO_2_ in Dulbecco's modified Eagle's medium (DMEM) supplemented with 10% foetal calf serum and 1% non-essential amino acids. To retain expression of stably transfected constructs, culture medium for CD8 cell lines was supplemented with 0.5 mg/mL G418. On-Target Plus siRNA oligos against PIKfyve and Vac14 were purchased from Dharmacon. A 2-hit protocol over 72 h was used. Briefly, HeLa cells were seeded in six-well plates at a density of 2.4 × 10^5^ cells/well. Cells were transfected with 40 nm of the corresponding oligo using oligofectamine (Invitrogen), 24 h later. After a further 24 h, cells were reseeded onto six-well plates at the same density or onto cover-lips at a density of 0.12 × 10^6^ cells/well and transfected again with 40 nM oligos for 24 h. Cells were then processed for western blotting and immunofluorescence as described below.

### Growth factor stimulation and cell lysis

Where indicated, cells were serum-starved for 16 h and then stimulated with 100 ng/mL EGF or hepatocyte growth factor (HGF). Cells were washed twice in ice-cold phosphate buffered saline solution and lysed in Nonidet P-40 lysis buffer (0.5% Nonidet P-40, 25 mm Tris/Cl, pH 7.5, 100 mm NaCl). Lysates were cleared by centrifugation, resuspended in 5× SDS-sample buffer and resolved by SDS-PAGE followed by immunoblotting for detection with a Licor Odyssey.

### Immunofluorescence

Cells were processed for immunofluorescence by fixation in 3% paraformaldehyde (PFA) in PBS, permeabilized with 0.2% Triton-X-100, blocked in 0.2% fish skin gelatin in PBS and incubated with antibodies in blocking buffer. Secondary antibodies were conjugated to either AlexaFluor 488, 594 or 633. Cover-slips were imaged using a Leica confocal SP2 AOBS (HCX PL APO CS 63.0 × 1.40 oil objective).

### HRP Internalization and Electron microscopy (EM)

Cells were incubated in 10 mg/mL HRP in serum-free medium at 37°C for 10 min, 1 h or 4 h. Subsequently, cells were washed three times in ice-cold PBS and fixed in 0.5% glutaraldehyde for 30 min. Cells were washed three times at room temperature with 0.1 m Tris-Cl pH 7.6, followed by incubation for 10 min in 0.1% diaminobenzidine (DAB, Sigma) in Tris buffer and then for 10 min in 0.1% DAB and 0.01% H_2_0_2_ in Tris buffer at room temperature in the dark. The reaction was terminated, by washing 3 × 5 min in Tris buffer, and then 2 × 5 min in PBS. Cells were then post-fixed in the dark on ice with 1% OsO_4_ in 0.1 m phosphate buffer pH 7.4 for 1 h. Subsequently, the cells were washed 3 × 30 mi in PBS, followed by 2 × 30 min in distilled water and then incubated in 5% uranyl acetate in 30% ethanol for 1 h. Samples were dehydrated by sequential 10-min ethanol washes in 30%, 60%, 70%, 80% and then twice in 100% ethanol, then infiltrated with a 1:1 ratio of resin to 100% ethanol for 30 min. The infiltration resin was then replaced with 100% resin and the flat-embedded samples were polymerized at 60°C for 2 to 3 days.

### Anti-CD8 uptake experiments

HeLaM CD8-CIM6PR or CD8-Furin cells were seeded onto cover-slips and treated with either siRNA or PIKfyve inhibitor. Cover-slips were then transferred to a well containing 3 mL ice-cold dihydroxybenzoate (DHB) (DMEM containing 25 mm Hepes and 0.2% fatty-acid free BSA) for 15 min. CD8-CIM6PR and CD8-Furin receptors at the cell surface were labelled by placing cover-slips, cell side down, upon a 100 μL drop of DHB containing 1 μg monoclonal anti-CD8 antibody at 4°C, for 1 h. Cover-slips were washed twice in ice-cold PBS, and then transferred to pre-warmed growth medium for the indicated time periods to allow uptake of the anti-CD8 antibody to occur. At the assay endpoint, cells were fixed and processed for immunofluorescence as described earlier.

### Shiga toxin uptake assay

The assay has been described previously (38). Briefly, HeLa cells were seeded onto cover-slips and treated with either siRNA or PIKfyve inhibitor as described earlier. Subsequently, cells were incubated on ice for 30 min with 2 μg/μL Cy3-STxB in DHB. The 0-min time-point was immediately fixed and the remaining cover-slips washed twice in ice-cold PBS, transferred to pre-warmed growth medium and returned to the incubator for the indicated time periods to allow uptake to proceed. At the assay endpoint, cells were fixed and processed for immunofluorescence as described earlier.

### Induction of autophagy and GFP-LC3 shift assay

HEK293A cells stably expressing GFP-LC3 were seeded onto 12-well plates and treated with siRNA or PIKfyve inhibitor as described above. To induce autophagy the growth medium was either replaced with fresh medium (fed) or cells were washed four times with Earl's buffered saline solution and then starved for 2 h in the final wash. Where indicated, Leupeptin was added to the starvation medium at a concentration of 0.25 mg/mL. Following starvation, cells were either fixed and processed for immunofluorescence or lysed in 1× SDS-sample buffer. After protein determination lysates were supplemented with 1 mm DTT and 0.1% bromophenol blue and resolved by SDS-PAGE for western blotting with polyclonal sheep anti-GFP antibodies, which were quantified using a Licor Odyssey instrument.
